# Life orientation teachers’ perspectives on intersectoral collaboration management of sexuality education

**DOI:** 10.4102/phcfm.v16i1.4703

**Published:** 2024-12-06

**Authors:** Zoe Pillay, Faniswa H. Mfidi

**Affiliations:** 1 Department of Nursing, Faculty of Science, Agriculture and Engineering, University of Zululand, Kwadlangezwa, South Africa; 2 Department of Health Studies, Faculty of Human Sciences, University of South Africa, Pretoria, South Africa

**Keywords:** adolescent, adolescent sexuality, reproductive health, implementation, intersectoral collaboration, LO teachers, sexuality education

## Abstract

**Background:**

According to the United Nations Fund for Population Activities, sexuality education (SE) requires building a multisectoral team and developing an operational plan. Hence, teaching comprehensive sexuality education (CSE) in collaboration with relevant stakeholders is a managerial approach to provide school-going adolescents with the knowledge and skills to make informed decisions that will safeguard their health.

**Aim:**

To report on Life Orientation (LO) teachers’ perspectives on intersectoral collaboration management of SE in secondary schools in KwaZulu-Natal (KZN) province, South Africa.

**Setting:**

Four selected secondary schools in the sub-district of uMhlathuze, KZN.

**Methods:**

The study employed a qualitative design. The population of this study comprised of 16 LO teachers who were selected by means of homogeneous sampling through focus group discussions. Emerging patterns were observed once the data were transcribed and subjected to thematic analysis.

**Results:**

The study revealed that schools were not being supported in the management of intersectoral collaboration in SE implementation in secondary schools in KZN province, South Africa.

**Conclusion:**

The results made it evident that more needs to be done to enhance and fortify intersectoral collaboration management to strengthen CSE in secondary schools.

**Contribution:**

The study recommended innovative and improved SE programmes and projects throughout the implementation and evaluation process to identify successes and promptly correct errors, to guarantee appropriate coordination of intersectoral collaboration management.

## Introduction

Life orientation (LO) and comprehensive sexuality education (CSE) aim to equip adolescents with the necessary knowledge, skills, attitudes and values to make informed decisions, create fulfilling relationships and productive lives. This goal aligns with the widely accepted belief that CSE encompasses a holistic approach that covers various aspects of an adolescent’s development, such as their physical, emotional, social and psychological well-being, and goes beyond merely sexual health.^1^ According to the Department of Basic Education (DBE), LO, a required basic life skills subject, is essential and needs to be improved in South African classrooms where children learn life skills for health and personal development.^2^ Hence, adolescents require age-appropriate, context-based, technological preference styled education and support to assist them to make informed and responsible decisions.^3,4^ Subsequently, beneficial approaches used in schools should be based on collaborations with diverse stakeholders. According to the Global Strategy for Women’s, Children’s, and Adolescents’ Health, there is a need for a meaningful change through intersectoral collaboration.^1^ Collaboration between several sectors to achieve shared objectives is known as intersectoral collaboration.^5^ These collaborations, especially when it comes to sexuality education (SE), bring together stakeholders from the social services, healthcare, education and civil society sectors to offer students comprehensive support. However, CSE support for intersectoral collaboration in secondary schools is a notable challenge.^6^ Understanding the relationships between the various stakeholders who are required to take action in a way that is more effective, efficient and sustainable is crucial in areas where intersectoral collaboration is required. South Africa’s policies regarding SE such as the Adolescent Sexual and Reproductive Health and Integrated School Health Policy have focused on a more all-encompassing and inclusive approach, emphasising intersectoral collaborations.^7,8^

Furthermore, according to the United Nations Fund for Population Activities (UNFPA),^8^ SE objectives require building a multisectoral team. Hence, teaching CSE in collaboration with relevant stakeholders is a managerial approach to provide school-going adolescents with the knowledge and skills to make informed decisions that will safeguard their health. Given that very little research has been conducted in schools in KwaZulu-Natal (KZN) in this regard, the researcher has taken note of this gap in knowledge.

Building a multisectoral management team and creating a strategic intersectoral collaboration approach are necessary for SE, according to UNFPA.^8^ Hence, intersectoral collaboration promotes the sharing of resources, knowledge and expertise, thereby reducing the duplication of effort and increasing productivity in a shorter space of time. Therefore, complementary to the South African Policies such as the Sexuality and Reproductive Health Policy, Integrated School Health Policy and DBE scripted curriculum, teaching CSE in partnership with stakeholders, many school-going adolescents will be able to make decisions that will protect their health by being well-informed of their rights to access high-quality CSE. Schools have a critical role in providing instructional programmes that reach many adolescents from a variety of backgrounds, providing opportunity to encourage adolescents to take cognisance of sexuality health.^9^ Consequently, an intersectoral collaboration management strategy engaging relevant stakeholders and teacher training can benefit the school-going adolescents as they gain expert knowledge and skills from various departments.^4^ The lack of teacher training can impede the implementation of CSE in educational settings.^10^ Moreover, although CSE programmes are taught all over the world, research indicates that they have not had much success in changing the sexual behaviours of adolescents. International studies confirm and concur with the current study that adolescents who do not receive adequate SE have numerous sexual partners and increased rates of sexual debuts as early as 15 years old are at risk of acquiring human immunodeficiency virus or acquired immunodeficiency syndrome (HIV or AIDS) and sexually transmitted infections (STIs),^11,12^ while findings in sub-Saharan Africa reveal that the first sexual encounters are from as young as 10 years old, adolescents have multiple concurrent sexual partners and 64% of the adolescents with HIV or AIDS.^13,14,15,16^ In 2022, there were around 7.6 million people living in South Africa who were HIV-positive, and the adolescent fertility rate were 97 births per 1000 girls.^17^ Moving forward, it is essential to address ongoing challenges by improving teacher training programmes and strengthening partnerships with external stakeholders. By doing so, South Africa can continue its efforts towards effective implementation of SE for the well-being of its young population. Furthermore, in order to track and measure the implementation and evaluate progress, monitoring and evaluation must be incorporated.^18^

### Research aim

This study aimed to describe the perspectives of the LO teachers regarding intersectoral collaboration management in SE implementation in secondary schools.

## Research methods and design

### Study design

The study employed a qualitative design owing to the descriptive and exploratory component to it.

### Study setting

The study setting was the four rural and urban secondary schools, a natural environment of the participant, which was the participants’ working environment in KZN, South Africa. The uMhlathuze Municipality reported 70 275 individuals who were HIV-positive in 2021 equating to 17.2% of the total population. This percentage is greater than the provincial levels of 15% and national levels of 11%.^19^ This implies that there is a greater demand for SE in this sub-district. Hence, the sub-district of uMhlathuze was selected.

### Sampling

Life orientation teachers from the four selected rural and urban schools in one health sub-district in the North Coast of KZN responsible for the implementation of CSE in secondary schools were recruited for the study. Two urban and two rural schools were selected for the study. There was a total of 16 participants in all four schools and all 16 participants were recruited. Homogeneous sampling was used to select the LO teachers. Inclusion criteria were that participants had to be teaching LO in the selected schools and present on the day that data were collected.

### Data collection

Permission was granted from the DBE, Department of Health (DoH), and District Hospital; thereafter, participants were approached to participate in the study. Sixteen LO teachers who met the criteria were given consent forms to sign. The purpose of the study and the participants’ rights concerning their participation were discussed. Data were collected using focus group discussions (FGDs) during the year 2022. The LO teachers responded to the research question on perspectives regarding intersectoral collaboration in SE in schools from which the themes, sub-themes and categories emerged as shown in Table 1. Each participant was given an opportunity to answer the questions. The researcher did not allow any participant to dominate the responses. The interviews were audio-recorded to capture all interview sessions. The researcher interviewed participants between the ages of 35 and 45 years across the four FGDs, of which seven were males and nine were females. A semi-structured interview guide was used. Focus groups were deemed appropriate for this study as they allowed the participants to share their perspectives, thoughts, views and experiences. The rationale for this approach was that they allowed for deeper and more thoughtful responses.

**TABLE 1:** Themes, sub-themes, and categories.

Themes	Subthemes	Categories
1. Implementation of sexuality education	Inclusion of sexuality education in school curriculum	Policy imperative Standardisation of content Current practices
	Views on intersectoral collaborations management	Ideal collaborations Empowering communities Youth involvement and support
	Effective implementation of comprehensive sexuality education	Communication channels Shared decision making Referral networks
2. Evaluation of the sexuality education	Perceived challenges	Socio-cultural issues Poor networking Inadequate resources
	Measures for improvement	Quality initiatives Ensuring continuity of service Monitoring and evaluation Strengthening capacity building

### Data analysis

Data were reviewed to identify common issues that recur such as intersectoral collaboration management of SE in secondary schools with verbatim supporting statements. The recordings were reviewed and verified by the second author. Data were transcribed verbatim, and coding was developed. Themes, sub-themes and categories were developed from individual quotes. Data from both phases were analysed using Flick’s^20^ thematic analysis steps and to identify common issues that recurred, and these were summarised in a narrative form. The analysis of texts pursued two goals: one was to reveal the statements or to put them in their context; the other goal was to reduce the original text by paraphrasing, summarising or categorising. Table 1 in the Results section shows the themes, sub-themes and categories.

## Trustworthiness in qualitative research

Trustworthiness is defined as validity that is applied to quantitative data.^21^ Credibility is a declaration of truth in interpretations of the data.^21^ The credibility of the study was enhanced by recording the interviews. According to Patton,^22^ dependability is concerned with the process of traceable documentation. The researcher enhanced the dependability of the study by maintaining an in-depth description of data. Confirmability means that there was confirmation that the data and findings are real.^22^ The researcher sought confirmation from the participants by conducting member checks. Transferability is referred to as transferring findings to another research.^22^ The researcher aimed to achieve transferability by providing in-depth descriptions to ensure replication of the study. Prior experiences, assumptions and beliefs of the researcher did not influence the data collection or research process.

## Ethical considerations

The study was granted ethics approval by the Higher Degree Ethics Review Committee of the DoH Studies, the Department of Basic Education, the KZN DoH and from the District Hospital in KZN (ethical clearance number: REC 012714-2020). The LO teachers signed a written informed consent and were informed of the right to withdraw without discrimination. Code numbers were used for identification to maintain anonymity and confidentiality. Data were stored verbatim on a laptop and will be destroyed permanently after 5 years.

## Results

Two themes with associated sub-themes emerged from the data collected from the four focus groups. In theme 1, namely, implementation of SE, the perspectives of the LO teachers on the implementation of SE are summarised. Three sub-themes emerged from the data: inclusion of SE in school curriculum, views on intersectoral collaborations, and effective implementation of CSE. In theme 2, that is, evaluation of SE, the LO teachers were given the opportunity to share their views regarding the factors that influence the implementation of SE in schools. They described challenges and proposed measures for improvement. Two sub-themes emerged from the data: perceived challenges and measures for improvement. It was important to determine the perspectives of the participants on the implementation of SE in secondary schools to determine the challenges and measures for improvement of the curriculum so that strategies could be identified and implemented for success. The categories are presented in Table 1.

### Implementation of sexuality education

Participants demonstrated understanding of the policies related to integrated childhood development that involve different sectors to be involved in various aspects of the health of a school-going child:

‘There is an integrated school health policy that talks about departments and other sectors working together with the schools to implement the policies. They would then know that they are obligated by law to integrate with the schools and share their expertise to promote health. To me this policy is not being implemented.’ (TFG4 P2 F)

The LO teachers described the current practices based on multiple sectors who visit schools to ensure continuity of sexual education as well as providing some support to the teachers:

‘We are collaborating with social workers as they come to our school to see the learners as our school is a boarding school, so they come and interview the learners from time to time.’ (TFG4 P1 M)

Most participants shared the view that intersectoral collaboration can work more efficiently if the government puts more effort into enforcing the implementation of this model. They presented ideas on the positive effects of different stakeholders participating in SE, as they believed that this was sensitive content, and the complexities of human behaviours require different disciplines to work jointly:

‘To me I would say that intersectoral collaboration is where departments come together, and team up discuss the various problems affecting the learners and then find solutions for each problem and then implement the solutions.’ (TFG2 P5 F)

‘I feel like the government should make it compulsory to make the department work together with the schools as part of their normal work routine.’ (TFG4 P2 F)

Participants believed that community and parents could have a greater role in SE if they had good understanding of what this involved. This could only be achieved if there is concerted effort from all stakeholders to increase awareness of this topic in the communities:

‘If we want any drastic change to take place in the community, then we must work hard by getting community leaders together and educating them about the benefits of the sexuality education curriculum to build a strong community as there are lots of bad reports on what is being taught in schools about this topic.’ (TFG1 P1 M)

There was a belief that every successful programme related to adolescent issues should allow their participation to increase the uptake. They were aware of the intent of the policies to empower youth. Youth deserved to be supported by giving them good access to various services:

‘When youth are given the opportunity to contribute to their own wellbeing then they become responsible for their actions. When the youth are integrated in adult health networks, they learn from those that have more experience.’ (TFG4 P3 F)

‘Like the Adolescent Youth Policy says, we need to empower the adolescents to get involved in policy formation and youth program development which the departments like the DBE and NGOs can assist with.’ (TFG3 P3 F)

The drive behind implementation of this subject is promoting overall health. Participants believed this required shared decision making in the course delivery, a sense of equal partnership and being competent to deliver this programme:

‘From the beginning every sector is involved in the decision making of the sexuality, education programme and they know what is expected of them and they all develop the programme together as an equal partner so they also feel a sense of belonging in the school and I think that this strategy will work well.’ (TFG4 P1 M)

Efficient referral systems are crucial in intersectoral collaborations to ensure continuity of care and sustainability. Emotional and physical health is key to these referrals, and teachers play a central role in supporting school-going adolescents in identifying the most relevant service or service provider for referral:

‘When we realise there is a problem with the learner then we call the parents and explain to the parent what the problem is then we advise the parent that help is available and free and that they can take the child to the relevant help.’ (TFG1 P3 M)

### Evaluation of the sexuality education

Sexuality education is not an easy topic to teach, especially when the generation gap between LO teachers and school-going adolescents is wider. Hence, participants thought that for certain topics, nurses would be better suited to present the content:

‘Students come from different cultures, and this can be a problem as many don’t want to listen to the teaching, whilst some feel shy, and some misunderstand the meaning of the words used. Cultural issues are major issues as this affects the way the learners understand the teaching, and this can lead to them getting confused.’ (TFG1 P3 M)

‘I would think that the clinics can come and show by demonstrating the certain topics that are hard to teach.’ (TFG1 P2 M)

Participants mentioned that it is a known fact that most schools lack resources. The LO subject needs audio-visuals and technology to make the content interesting and well understood:

‘We need more LO teachers. We are only 3 here and have to teach so many classes. We need more staff employed to be LO trained to assist us with the workload.’ (TFG4 P2 F)

The focus of this sub-phase is on intersectoral collaborations. However, the idea needed to be contextualised. Hence, views were elicited regarding how the implementation of SE in schools could be improved. Participants acknowledged the importance of ensuring quality of learning materials and teaching practices. They indicated a need for collaborative supportive visits and learner-centred content:

‘I think that to add quality to the programmes, quality assurance people should come and monitor the sex education curriculum programme to see if it is a high-quality sex education is delivered to the learners.’ (TFG1 P4 F)

‘CSE should be learner-centred according to needs and age group and can be made exciting by allowing other organisations to assist in the teaching as this will enhance the knowledge that the learners and us will gain.’ (TFG3 P2 F)

Participants expressed the need to ensure continuity of quality service to school-going adolescents. Funding to be reviewed and adjustments made where necessary for various interventions:

‘Arrangements need to be made with the government for funding of the programme as there may be times when events and festivals need to be planned and implemented so funding is needed for that.’ (TFG3 P1 F)

‘I think that schools need to embrace partnering with various departments to succeed in carrying out quality education to learners. Some learners like to learn in different styles and by bringing in other departments to assist in teaching this really helps a lot.’ (TFG3 P2 F)

Having robust monitoring and evaluation systems would ensure effective implementation of CSE. Monitoring what is working and not is an essential tool for getting feedback:

‘Departments needs to take responsibility of schools and visit regular to find out about the success or failure of CSE teaching.’ (TFG3 P1 F)

‘The departments need to get involved with the schools in a way that they work together and not separately. They need to communicate with each other and discuss the needs of the learners.’ (TFG2 P2 M)

Participants appeared more than willing to take some responsibility for teaching this subject effectively. They recognised their shortcomings and indicated the need for training to build capacity in the content and presentation of this subject. Various sectors could play a role in this initiative:

‘I suggest that the principal and us should connect with various sectors to explore the latest advances for training and development program and making these available to all of us teachers for professional and personal growth.’ (TFG1 P2 M)

‘The sex education curriculum should be taught to each of us, and we should have a certificate in it before even attempting to teach the subject as it contains sensitive topics that not everyone wants to teach.’ (TFG1 P1 M)

## Discussion

This study sought to describe the LO teachers’ perspectives on intersectoral collaboration management of SE in secondary schools. Therefore, it was important to get an in-depth understanding of the current processes involved in the execution of SE in schools and how the LO teachers perceive and experience the implementation of the SE curriculum in secondary schools. The main themes that arose from the findings were the implementation of SE and evaluation of SE. Policy imperatives, current practices, shared decision-making, empowering communities, youth involvement and referral networks were the highlighted categories under the implementation of SE in secondary schools. The goals of LO and CSE are to provide adolescents with the information, abilities, attitudes and values they need to make wise decisions. This objective is consistent with the widely held view that CSE encompasses a holistic approach that goes beyond merely covering sexual health and covers various aspects of an adolescent’s development, such as their physical, emotional, social and psychological well-being.^23^

Data showed that there was a high level of knowledge of the policies among LO teachers. Recent studies^6,24^ show that understanding policies improves how SE is implemented and sustained.

However, there were challenges with intersectoral collaboration support of CSE in secondary schools. In areas where intersectoral collaboration is mandatory, it is critical to recognise the relationship between the different stakeholders who have been mandated to take actions in a way which is more effective, efficient and sustainable.^6^ The results from the LO teachers demonstrate that the curriculum should be made available and accessible to all stakeholders. Participants believed the curriculum could be modernised and standardised to ensure that the material is understood by all relevant sectors.^25^ Life orientation teachers confirmed that there was a lack of visits from relevant stakeholders. However, data from all schools revealed that there was less input from the relevant stakeholders. The exception is that only social workers came at regular intervals to visit a particular boarding school. It should be noted that these visits were not consistent to other schools.

Recent studies^4,26^ found that technologies provide students with freedom to access digital knowledge according to their learning styles. This suggests that multimedia strategies such as the use of various media, websites and community projects can be implemented in CSE in the schools. By expanding multiple teaching spaces, school-going adolescents can deepen their understanding, develop lifestyle skills and make them better prepared for the real world. However, this study contends that mere talk about the use of technology-supported teaching is not a guarantee that school-going adolescents would change their risky behaviours. There are multiple factors that need to be considered to make SE effective.^27^

Life orientation teachers concur that there was a lack of visits from the other departments to assist, support or guide them in teaching difficult topics that need experts in the field of SE to demonstrate and teach. In addition, school-going adolescents confirm that there was an absence of visits from the relevant stakeholders that could have been a support system for them in learning and understanding difficult topics. They also believed that if the experts in the field taught the topics, they may have a better understanding of the subject. Hence, the challenge of inconsistent and inequitable schools’ coverage needs to be addressed. This infers that legislative requirements for the relevant stakeholders to follow are providing school health services and school-based health promotion visits. This is consistent with previous studies^9,28^ which found that shortage of workforce was a challenge and a barrier in service delivery to schools.

The study recognises that it is imperative for different sectors to engage in discussions on how they can improve collaboration management, coordination and identifying solutions to the barriers that prevent the implementation of CSE in schools. Life orientation teachers indicated that the relevant stakeholders must assume responsibility for schools and conduct routine assessments to see if the CSE programme implemented in schools is effective or not. The study assumes that monitoring and evaluation are only effective if the data gathered from different sectors are considered. This is in line with other studies^8^ which concur that monitoring and evaluation are an essential process that helps to identify what is working and what is not, and to make informed decisions on how to improve their programmes. Additionally, the National Adolescent and Youth Health Policy (NAHYP) states that monitoring and evaluation must be included to track and measure the implementation of the NAHYP and to assess progress.^18,29^

The relevant stakeholders should ensure that innovative and improved SE programmes, projects, events and festivals are evaluated throughout the implementation process in order to determine successes and correct errors promptly, thus ensuring the suitable integration of intersectoral collaboration management.^30^ The relevant stakeholders should ensure that collaboration and teaching are based on evidence-based practice that has been proved successful. The relevant stakeholders should initiate and integrate intersectoral collaboration management of SE in schools as they are professionals in the field of health.

Further research should be conducted in the following areas: LO teachers’ perceptions of mutuality between departments and a comparative study between rural and urban secondary schools to explore CSE implementation.

## Limitations

The study focussed only on CSE implementation at one KZN sub-district, and therefore, a similar study in another sub-district could produce different findings. However, in qualitative research, the researchers do not seek to generalise the findings. In this study, the researcher sought an understanding that might prove useful in other situations, but that also may not apply.

## Strengths of the study

The results would harness strengths from various sectors to develop comprehensive educational approaches that would empower adolescents on all matters pertaining to sexuality and sexual behaviours.

## Conclusion

The study provided evidence of the perspectives of LO teachers on SE implementation in secondary schools. Including parental and community involvement in the research would have increased the understanding of the problems associated with parental and community collaborations. The study found that there was some form of intersectoral collaboration between the relevant stakeholders. However, the strength of this collaboration could not be determined. The researcher also makes references to the study’s significance, appropriateness and relevance considering the need for an intersectoral support in schools. Therefore, research on CSE cooperation support ought to be viewed as an endeavour to enhance and fortify the use of CSE in secondary education. The evidence produced by the study contributed to the recommendations for the intersectoral collaboration approach.
